# H3K36 Methylation in Neural Development and Associated Diseases

**DOI:** 10.3389/fgene.2019.01291

**Published:** 2020-01-09

**Authors:** Mattia Zaghi, Vania Broccoli, Alessandro Sessa

**Affiliations:** ^1^Stem Cell and Neurogenesis Unit, Division of Neuroscience, San Raffaele Scientific Institute, Milan, Italy; ^2^Concilio Nazionale Delle Ricerche (CNR), Instituto di Neuroscienze, Milan, Italy

**Keywords:** H3K36, histone methyl transferase (HMT), neurodevelopment, SET – Su(var)3–9 and ‘Enhancer of zeste’, DNA metyltransferases (Dnmts)

## Abstract

Post-translational methylation of H3 lysine 36 (H3K36) is an important epigenetic marker that majorly contributes to the functionality of the chromatin. This mark is interpreted by the cell in several crucial biological processes including gene transcription and DNA methylation. The homeostasis of H3K36 methylation is finely regulated by different enzyme classes which, when impaired, lead to a plethora of diseases; ranging from multi-organ syndromes to cancer, to pure neurological diseases often associated with brain development. This mini-review summarizes current knowledge on these important epigenetic signals with emphasis on the molecular mechanisms that (i) regulate their abundance, (ii) are influenced by H3K36 methylation, and (iii) the associated diseases.

## Introduction

Post-translational modifications of histones are one of the most relevant and best studied mechanisms of epigenetic control used by the cell. Among them, acetylation and methylation of lysine (K) within N-terminals (or tails) of histones (mainly H3) have risen to fame by defining the topological distribution of active and inactive regions in the genome.

High levels of methylation are described at H3K4, K9, K27, K36, K79, each of them having different roles in determining functional features of the chromatin. Methylations of H3K9 and H3K27 are generally described as repressive markers, whereas H3K4, H3K36, H3K79 usually designate regions of active chromatin (Black et al., 2012).

Recently, H3K36 methylation has gained a lot of interest because of its role in several key cellular processes and subsequently many human pathologies. H3K36 methylation has been found to be involved in transcription and splicing regulation (Wang et al., 2007; Edmunds et al., 2007; Pradeepa et al., 2012; Shin et al., 2014), DNA damage response (Pai et al., 2014; Pfister et al., 2014), DNA methylation and dosage compensation (Larschan et al., 2007) among others.

In contrast to acetylation, where only one acetyl group can be inserted, lysine methylation can occur on three different levels, where one, two, or three moieties (mono-, di-, or tri-methylation) can be added to the residue. Each state possibly represents a different epigenetic mark within the so-called epigenetic ‘code’ (Turner, 2007). H3K36 mono-methylation (H3K36me1) is considered an intermediate modification, apparently without any significant role, whereas different role and distributions have been demonstrated for both di- (H3K36me2) and tri-methylation (H3K36me3); H3K36me3 is present in gene bodies while H3K36me2 is found in intergenic regions, both within lightly packed chromatin. Mass spectrometry analyses of histone modifications have estimated the relative abundances across different organs, revealing that the H3K36 either un-methylated or di-methylated in K36 are the most abundant spanning between 20% to 50% each while the mono-methyl form and the completely saturated proteins are rare with H3K36me3 that reach the 5% of total H3 (Yu et al., 2016; Li et al., 2019). Notably, H3 labeled with K36me2 showed high turnover rate compared to the other forms (Zee et al., 2010).

Much has been done in recent years to investigate the specific molecular players involved in H3K36 methylation. Still, a lot of questions remain open regarding the biological meaning of these marks, conveyed by their specific location, as well as the molecular machinery involved in their generation, removal, or interpretation.

In this review, we will provide an overview of recent discoveries and introduce the concept that H3K36 methylation might represent the epigenetic master regulator of euchromatin function. We will put emphasis on developmental disorders in which the alteration of these epigenetic marks has been shown lead to neurological symptoms.

## Enzymatic Control of H3K36 Methylation

**Figure 1:**
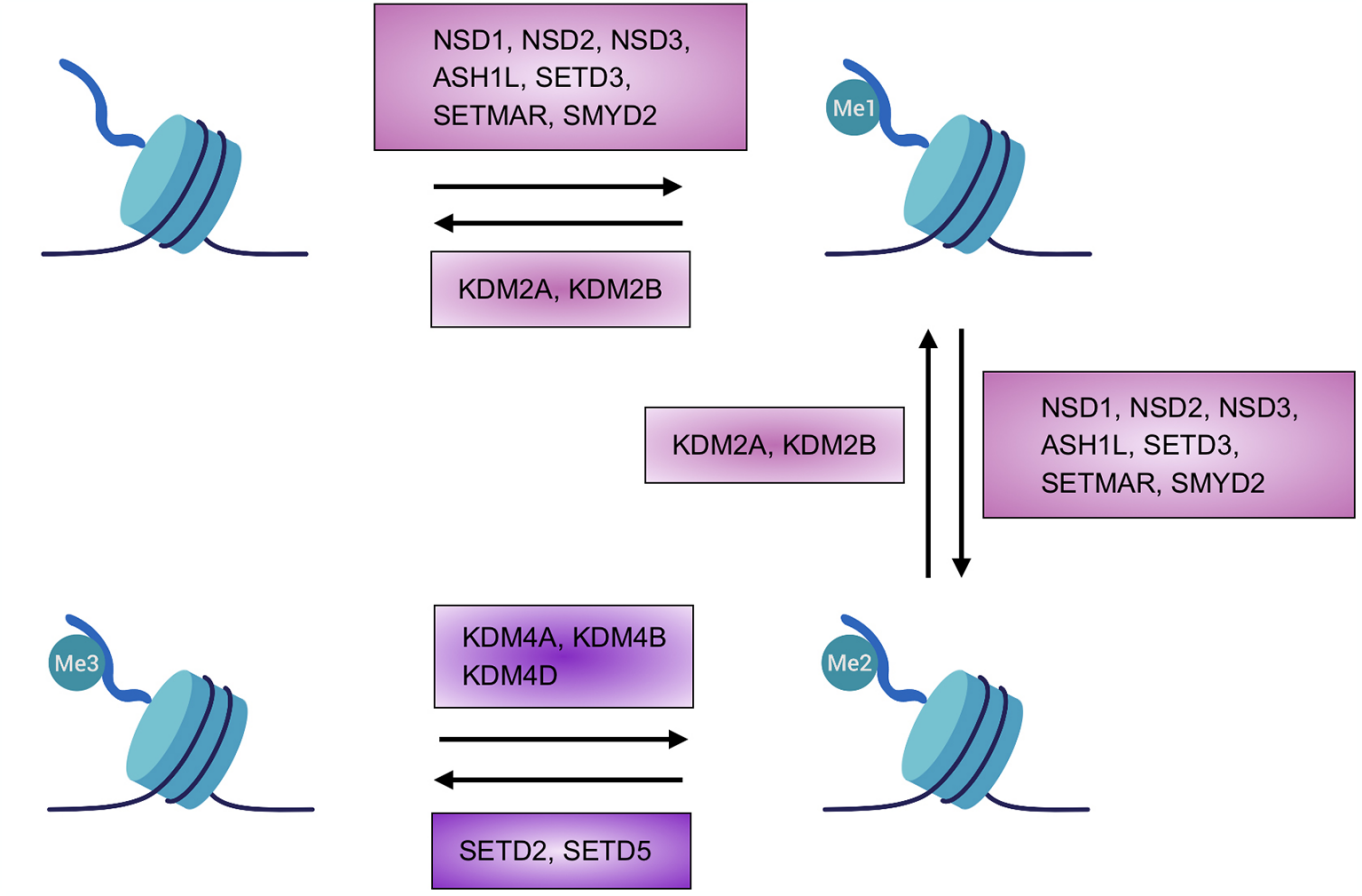
Homeostasis of H3K36 methylation. Depiction of the transition between the different states or chromatin marks of lysine 36 of histone H3. Known enzymes found in mammals able to add or removemethyl groups are highlighted. Created with BioRender.com.

H3K36 methylation is regulated by several mechanisms that control the distribution of the marks as well as the transduction of their biological meaning. The different molecular players (**Supplementary Table S1**), which are either highly specific for H3K36 or shared with other residues, may be grouped into three categories; the so-called “writers,” which are enzymes that catalyze the methylation reaction, and the “erasers,” which perform the opposite function and catalyze the removal of methyl groups, together maintain the right homeostasis between the two states (**Figure 1**). The third category is comprised by the “readers,” which are proteins that are able to recognize and interact with specific modifications, possibly interpreting its message. Most of the writers and erasers of lysine methylation are also readers of the same and/or other modifications, however, there are other proteins for which H3K36 methylation acts as a docking signal that regulates downstream functions or pathways.

### Writers

H3K36 writers are histone methyltransferases (HMTs) that contain a SET (Suppressor of variegation 3-9, Enhancer of Zeste, and Trithorax) domain and use S-adenosylmethionine to add methyl groups to histones (or other proteins) (Dillon et al., 2005). H3K36 methylation is an evolutionary conserved process, however, in yeast, only one enzyme—HMT Set2 (SET domain containing protein-2)—carries out each step from mono- to tri-methylation (Krogan et al., 2003; Xiao et al., 2003); in higher eukaryotes, the different steps required to reach full methylation are catalyzed by several enzymes. Most of these enzymes methylate up until a state of di-methylation whereas only two members are able to catalyze the last step to achieve the tri-methylated state (**Figure 1**).

NSD1 (nuclear receptor SET domain-containing protein 1), initially described as nuclear steroid hormone receptor (Huang et al., 1998), has been shown to catalyze H3K36 and H4K20 methylation (Rayasam et al., 2003). Recent studies have restricted its specificity to the di-methylation of H3K36 thanks to experiments performed on purified nucleosomes (Li et al., 2009; Qiao et al., 2011). Notably, its absence results in a reduction in all three states of histone methylation, demonstrating the critical role of NSD1 activity even in the tri-methylation step that is carried out by other enzymes, probably as supplier of the right amount of substrate for subsequent reaction (Lucio-Eterovic et al., 2010).

Of the same family, NSD2, first identified due to its involvement in chromosomal deletion, causes Wolf-Hirschhorn syndrome (WHS) when mutated (Stec et al., 1998). NSD2 exists in two main isoforms, one catalytically active and the other without the SET domain resulting in an inactive form of the enzyme (Stec et al., 1998). As for NSD1, NSD2 can potentially catalyze the mono- and the di-methylation of H3K36 (Martinez-Garcia et al., 2011; Morishita et al., 2014), although recent studies suggest its preference to perform di-methylation instead of mono-methylation (Qiao et al., 2011; Poulin et al., 2016). The mechanism of action of NSD2 is based on the recognition of regions in which H3K36 is already di-methylated thanks to its PWWP (Pro-Trp-Trp-Pro) domain, which stabilizes the protein on the chromatin and directs methyltransferase activity to adjacent regions of chromatin. Thus, in contrast to NSD1, NSD2 functions more as a propagator of methylation rather than a *de novo* methyltransferase (Waggoner et al., 2005; Kuo et al., 2011; Martinez-Garcia et al., 2011; Morishita et al., 2014; Sankaran et al., 2016).

NSD3 was identified by searching genes with sequence similarity to NSD1 and NSD2 (Angrand et al., 2001; Stec et al., 2001). NSD3 has been found to be expressed in the brain, heart, and skeletal muscle, and to a lesser degree in the liver and lung (Angrand et al., 2001). Three protein products have been associated with NSD3: long, short, and WHISTLE (WHSC1-like 1 isoform 9 with methyltransferase activity to lysine). The long (1437 aa) and short (645 aa) isoforms share the same amino terminal sequence, but the short form lacks the catalytically active SET domain and is only able to bind methylated H3K36, thanks to its PWWP domain (Stec et al., 2001; Vermeulen et al., 2010; Wu et al., 2011). WHISTLE is the shortest isoform (506 aa) that includes an active SET domain. WHISTLE activity, however, is associated with methylation of H3K4 and H3K27 and thus transcriptional repression (Kim et al., 2006; Allali-Hassani et al., 2014).

Outside of the NSD family there are other enzymes that have been described as able to methylate H3K36 as mono- and di-methyltransferases. Activity has been described for ASH1-like (ASH1L), SETMAR (SET domain and mariner transposase fusion gene-containing), SETD3, and SET and MYND domain-containing 2 (SMYD2). These enzymes do not show high specificity, indeed recognition of other substrates has been demonstrated (Lee et al., 2005; Brown et al., 2006; Tanaka et al., 2007; Abu-Farha et al., 2008). As an example, SMYD2 and ASH1L can methylate H3K4, with the latter able to modify also H4K20 (Huang et al., 2006; Gregory et al., 2007).

Up until recently, the only enzyme known to tri-methylate H3 was SETD2 (Edmunds et al., 2007). SETD2 contains a SET domain and, as for the yeast ortholog, Set2, retains the ability to interact with the large subunit B1 of RNA polymerase II (RNAPII) (Kizer et al., 2005). Intriguingly, the association of SETD2-RNAPII is critical both for HMT activity of SETD2 and for the correct travelling of RNAPII during the elongation phase (Wagner and Carpenter, 2012; McDaniel and Strahl, 2017). However, residual H3K36me3 levels have been reported upon genetic loss of Setd2 in multiple systems (Hu et al., 2010; Pfister et al., 2014; Zhang et al., 2014).

Indeed, we were able for the first time to describe that another SET containing protein, SETD5, can methylate H3K36, both *in vitro* and *in vivo*, up to the tri-methylated form (Sessa et al., 2019) (**Figure 1**). We verified that SETD5, as SETD2, has the capacity to facilitate transcriptional processivity even without direct binding to RNAPII (Sessa et al., 2019), however, further work is needed to clarify the characteristics of this “new” H3K36 HMT.

### Erasers

To maintain a proper control and balance between methylated/un-methylated states, enzymes (KDMs, Lysine histone demethylases) that can remove post-translational modifications from specific residues, including H3K36, are necessary. These enzymes belong to Jumonji family since they contain an essential JmjC catalytic domain (Whetstine et al., 2006; Crona et al., 2013).

In yeast there are two proteins which are able to erase H3K36 methylation, Jhd1 (KDM2) and Rph1 (KDM4) (He et al., 2008; Crona et al., 2013), while in mammals, the demethylation of H3K36 is performed by two protein families, KDM2A/2B (orthologs of Jhd1) and KDM4A/4B/4D (Rph1) (**Figure 1**). Specifically, H3K36me1-me2 demethylation is performed by KDM2A/2B (Tsukada et al., 2006) while KDM4A/4B/4D has specificity for H3K36me3, but is also able to act on H3K9me2/me3 (Cloos et al., 2006; Klose et al., 2006).

### Readers

As for every histone modification, H3K36 methylation is essential to orchestrate nucleosome functions within chromatin. In order to execute this function, H3K36 signatures must be recognized by chromatin cofactors. In the case of H3K36me, reader proteins often contain one of the following domains: tudor, PWWP, or chromodomain (Li et al., 2019). As mentioned before, many epigenetic writers may also function as readers to ensure either the completion of residue methylation or the spread of a different methylation state to nearby residues.

In addition to the already cited roles of the NSD proteins, many other proteins contain PWWP domains, including those able to methylate DNA (DNMT3A and 3B), those involved in DNA repair (MSH6, LEDGF) and the H3.3K36m3 specific reader, ZMYND11 (Li et al., 2019). PHF1 and PHF19 contain tudor domains and are responsible for facilitating the entrance of Polycomb repressive complex 2 (PRC2) in H3K36me marked regions (Cai et al., 2013). The H3K36me3 reader, MRG15, contains a chromodomain and can interact with other epigenetic modifiers, such as the histone demethylase KDM5B, to promote H3K4me3 removal from intragenic regions (Hayakawa et al., 2007).

## Biological Roles of H3K36 Methylation

Experimental evidence obtained from different experimental systems suggests that H3K36 methylation is a major central epigenetic modification that orchestrates the function of euchromatin. Indeed, while euchromatin has been widely associated with histone acetylation, which is physically associated with chromatin opening, H3K36me has a clear role in defining important regions of active chromatin.

**Figure 2:**
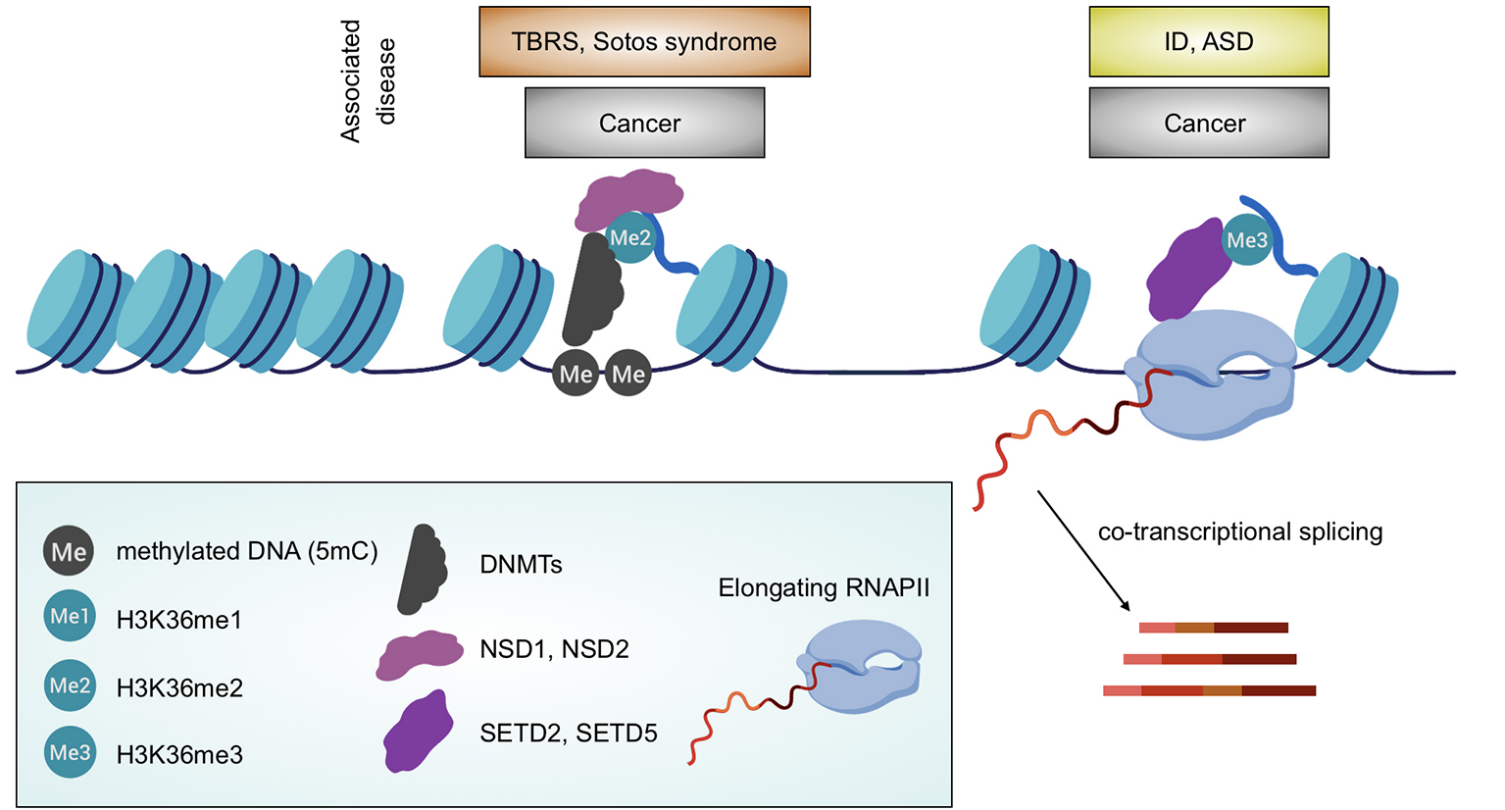
Association between H3K36 methylation and biological processes. Depiction of the linkage between H3K36 HMTs with either DNA methylation distribution or transcription including co-transcriptional processes (RNA splicing is shown). Associated diseases are also highlighted. Created with BioRender.com.

This concept is especially supported by the role of H3K36 methylation in the regulation of transcription and co-transcriptional processes (Wang et al., 2007; Edmunds et al., 2007; Pradeepa et al., 2012; Shin et al., 2014; Sessa et al., 2019) and, by association, with DNA methylation (Baubec et al., 2015; Weinberg et al., 2019) (**Figure 2**). Furthermore, H3K36 methylation has been demonstrated to be essential for regulated segregation of heterochromatin regions, via the control of CBP/p300 localization (Cabianca et al., 2019).

In this section, we will dissect the molecular role of this epigenetic modification, and thus the biological consequences in having or not the H3K36me for a cell.

### H3K36 Methylation and Transcription

H3K36me3 is found in the body of transcriptionally active genes, a genomic landscape where finely tuned and intimately interconnected mechanisms occur. In these regions, RNA elongation, splicing, and inhibition of the generation of spurious intragenic transcripts are coordinated to ensure effective transcriptional processes. The relationship between H3K36me and transcription was demonstrated first because it was found that Set2, thanks to its SRI domain, is able to interact with the phosphorylated CTD (C-terminus domain) of the active RNAPII. The abrogation of this interaction via the removal or mutation of the SRI domain causes not only a strong alteration in transcriptional elongation, but also disrupts the methyltransferase activity of Set2 (Wagner and Carpenter, 2012; McDaniel and Strahl, 2017). The relationship between RNAPII travelling and H3K36me is important also to limit the initiation of aberrant intragenic transcription along active genes, which in yeast relies upon local deacetylation activity (Carrozza et al., 2005) and remain elusive in mammals (Carvalho et al., 2013). Moreover, inclusion/exclusion of exons has been associated with intragenic H3K36me3 levels in gene bodies through the recruitment of H3K36me3 readers (MRG15 and ZYMIND11) that directly modulate the activity of splicing factors (Luco et al., 2010; Guo et al., 2014).

In higher eukaryotes, both H3K36me2 and me3 have been described to regulate transcription. In particular, H3K36me2 in gene bodies is enriched in expressed genes, positively correlating its presence with transcription levels, even though it seemed not essential (Bannister et al., 2005; Kuo et al., 2011). NSD2 overexpressing tumor cells showed a de novo oncogenic program caused by the re-location of H3K36me2 (Kuo et al., 2011). The safeguard of transcription and co-transcriptional processes, instead, have been associated with SETD2 activity, the only known HMT able to achieve H3K36me3 (Luco et al., 2010) (Wagner and Carpenter, 2012). Recent evidence from our group suggest that the paralog protein, SETD5, is able to tri-methylate H3K36 (Sessa et al., 2019). Accordingly, we have shown that a low level of *Setd5* in the murine nervous system causes a decrease of H3K36me3, which results in global perturbation of the transcriptome. This was particularly evident for highly transcribed genes, which displayed reduced transcriptional processivity and a higher percentage of aberrant splicing events (Sessa et al., 2019). Thus, H3K36me3 dysregulation, due to the impairment of either SETD2 or SETD5, underlines the central role of H3K36me3 in defining the epigenetic landscape in gene bodies that controls the stability and activity of euchromatin. As such, fine tuning H3K36me distribution and quantity is vital for the cell to such an extent that even a slight change in methylation is detrimental on many levels, as testified by the related occurrence of many diseases (see later).

### H3K36 Methylation and DNA Methylation

Recent work has demonstrated how the process of DNA methylation is tightly regulated and linked with both H3K36me2 and me3 (Baubec et al., 2015; Morselli et al., 2015; Weinberg et al., 2019). Experimental evidence suggests that the binding profile and consequently the activity of the DNMTs is influenced by the presence of H3K36me2/3, findings in line with their activity as H3K36me reader. Using mouse embryonic stem cells, it has been demonstrated how the activity of SETD2, and thus the level of H3K36me3, are essential for the localization and activity of DNMT3B (Baubec et al., 2015). Indeed, both *Setd2* KO and mutation of the PWWP domain in DNMT3B impaired DNA methylation (Baubec et al., 2015). More recently, it has been proposed that the portion of the genome that is H3K36me2 positive is highly enriched (75%) in CpG methylation (Weinberg et al., 2019). Furthermore, these regions are also marked by H3K4me1 and H3K27ac, highlighting how H3K36me2 defines active regions of euchromatin, especially in intergenic regions. The authors in this work also specified that DNMT3B activity depends on H3K36me3 whilst DNMT3A is associated with regions enriched in H3K36me2, usually marking intergenic regions, thus demonstrating that different H3K36 methylation states precisely control methylation levels in different chromatin regions. Nsd1/2 knock-out in embryonic stem cells causes a total depletion of H3K36me2 within intergenic regions and consequent loss of binding and activity of DNMT3A which instead is repositioned across DNMT3B domains. Taken together, these findings demonstrate that there is a fine crosstalk between DNA and H3K36 methylation, which orchestrates the distribution and activity of different DNA methyltransferases in euchromatin.

## H3K36 Methylation in Neurodevelopmental Diseases

Not surprisingly, alterations in the H3K36 methylation levels are associated with different athologies, including many types of cancer and neurological disorders of developmental origin (**Figure 2**). From a genetic view point, these alterations may be due to either mutations in KTMs or KDMs, or even due to the substitution of the K36 residue itself (K36M), or nearby residues (e.g. G34V/R) creating so-called onco-histones (Nacev et al., 2019). Additionally, some diseases may be linked with H3K36me, despite no differences in the level of markers, because of an impairment in the downstream interpretation of these epigenetic signals, e.g. due to mutations in genes encoding for H3K36me readers.

Furthermore, the analysis of animal models deficient in specific H3K36 HMTs testify the importance of having the correct levels of the different H3K36me modifications. Indeed, knock-out (KO) mouse models of NSD protein 1 and 2 are both lethal, either very early during development (at embryonic day (E) 6.5) in the case of *Nsd1* (Rayasam et al., 2003), or at post-natal day (P) 10 for *Nsd2* (Nimura et al., 2009).

Lethality is also true for KO of the two H3K36 tri-methyltransferases, where the full *Setd2* KO is viable until E10.5-E11.5 (Hu et al., 2010), and the Setd5 KO is lethal at E9.5 (Osipovich et al., 2016). Lastly, also the H3K36me erasers have emerged as important for neural cell development; in fact, KDM proteins have been found essential for neuronal differentiation, survival, and either the expression or repression of specific genes (e.g. expression of Bdnf, repression of Gfap) (Cascante et al., 2014; Sudo et al., 2016), while KDM8 has been associated with proliferation of Schwann cells controlling the expression of cell-cycle relevant genes (Fuhrmann et al., 2018).

In the following paragraphs, we will describe the different pathologies associated with the aforementioned cellular processes, with a focus on their neurological implications.

### H3K36me Pathologies Associated With Splicing Regulation

The central nervous system is probably the site where the greatest number of alternative splicing events occur; this is even more true in neurons where the difference between each subtype is frequently determined by the expression of different splice isoforms of several key genes (Furlanis et al., 2019).

Mutations in SETD2 gene are associated with cases of autism spectrum disorders (ASD) (Iossifov et al., 2014; Lumish et al., 2015) and with “Sotos like syndrome,” an overgrowth disease with cognitive impairment (Luscan et al., 2014). A growing number of evidences link haploinsufficiency of SETD5 to cases of intellectual disability (ID), ASD, and 3p25.3 microdeletion syndrome (Rauch et al., 2012; Kellogg et al., 2013; De Rubeis et al., 2014; Grozeva et al., 2014; Kuechler et al., 2015; Szczałuba et al., 2016; Parenti et al., 2017). Analysis of *in vitro* and *in vivo* loss-of-SETD5-function models revealed the importance of the correct level of H3K36me3 for the correct structural and functional maturation of the central nervous system (Sessa et al., 2019). Indeed, pathological phenotypes seem to be caused by defects in RNA elongation/RNAPII processivity (Deliu et al., 2018; Sessa et al., 2019) that cause a massive deregulation of co-transcriptional processes, including splicing events (Sessa et al., 2019).

Both *NSD1* and *NSD2* haploinsufficiency lead to neurodevelopmental syndromes, namely Sotos syndrome and WHS, respectively (Kurotaki et al., 2002; Nimura et al., 2009). Strikingly, a percentage of WHS patients is mutated in the transcription elongator factor gene *NELFA*, inducing a decrease in elongation rates (Yamaguchi et al., 1999). Thus, the association between SETD5-H3K36me3-RNA balance is instructive for the importance of this epigenetic modification in the correct structural and functional maturation of the central nervous system. However, the interconnection of H3K36me levels and co-transcriptional events has probably a broader impact since other devastating diseases, such as several types of cancer, display a correlation between low H3K36me3, caused by mutations in either *SETD2*, or *SETD5*, or *H3* and an impairment in transcriptional fidelity and co-transcriptional mechanisms, such as RNA processing and stability, chimeric RNAs, aberrant cryptic transcripts etc. (Fontebasso et al., 2013; Bueno et al., 2016; Papillon-Cavanagh et al., 2017).

### H3K36me Pathologies Associated With DNA Methylation

Tatton–Brown–Rahman syndrome (TBRS) (Tatton-Brown et al., 2014) and Sotos syndrome (Kurotaki et al., 2002) share many pathological phenotypes, which may be due to the relationship between H3K36 methylation and DNA methylation.

TBRS is caused by mutations in *DNMT3A* gene, with a proportion of mutations having been found within the PWWP domain which, as described before, allows for the reading of H3K36 methylation. This syndrome is described as an “overgrowth syndrome,” which is characterized by distinctive facial appearance, tall stature, mild to severe cognitive deficits, which result in behavioral/psychiatric issues, and some combination of traits typical of ASD (Tatton-Brown et al., 2018).

*NSD1*-associated Sotos syndrome presents with overgrowth as the main phenotype during the first years of life, with distinctive facial traits and developmental delays characterized by intellectual disability (Waggoner et al., 2005). Individuals with Sotos syndrome can have mild to severe mental retardation, causing behavioral abnormalities and social struggle (Sarimski and Psychologist, 2003). Interestingly, a strong and generalized decrease in DNA methylation levels has been found in blood samples of Sotos syndrome patients (Choufani et al., 2015), as was found in TBRS patients.

The concept that DNA methylation is essential for mammalian brain development and associated human pathologies, notably Rett syndrome (Amir et al., 1999), is not new and has extensively been described (Lister et al., 2013). What is lesser known, however, is how the DNA methylation is finely regulated. Both Sotos and Tatton–Brown–Rahman syndromes, previously discussed, are caused by completely different mutations and yet have major clinical and molecular phenotypes in common; this supports the importance of the intimate and profound connection between H3K36me, distribution of DNMTs and DNA methylation (**Figure 2**).

Again, even in this case, cancer biology further supports this observation since cases in head and neck squamous cell carcinomas present mutations in NSD1, within or close to the SET domain, or K36M substitution and remarkable DNA hypo-methylation (Papillon-Cavanagh et al., 2017).

## Conclusions

Histone post-translational modifications of H3K36 define at least two widespread epigenetic marks (H3K36me2 and me3) with an extensive role in determining the chromatin landscape and in directing complex biological processes. Some of these best characterized processes include: (i) the control of transcriptional fidelity, meaning the correct execution of transcription and other strictly associated mechanisms, (ii) the control of DNA methylation, (iii) DNA repair, and (iv) the interplay with other epigenetic marks. However, the complexity of H3K36me regulation, including the discovery of a new HMT, and the elusive nature of H3K36me2 decoration on the genome suggest that many aspects still need to be elucidated in the future. Moreover, less famous and studied modifications of the same residue, as in the mono-methyl form as well as the acetylated version (H3K36ac) (Morris et al., 2007), may hide some neglected functional implications. Since the high correlation between H3K36 modifications and human diseases, more work is required to complete the picture. The community will benefit from the interconnection of the different experimental approaches of biochemistry, structural biology, new *in vitro* and *in vivo* model systems as well as from a thorough clinical observation coupled with molecular profiling of patients.

## Author Contributions

All authors have contributed to the conception of this article and approved it for publication.

## Funding

This work was supported by the Italian Ministry of Health (GR-2013-02355540 to AS).

## Conflict of Interest

The authors declare that the research was conducted in the absence of any commercial or financial relationships that could be construed as a potential conflict of interest.

## Supplementary Material

Supplementary Table 1 Genes/proteins cited in this mini-review with official names and aliases.
